# Gastrointestinal Fistulas—What Gastroenterologists Need to Know in 2025

**DOI:** 10.1155/cjgh/6210421

**Published:** 2025-08-13

**Authors:** Monjur Ahmed

**Affiliations:** Department of Medicine, Division of Gastroenterology and Hepatology, Thomas Jefferson University Hospitals, Philadelphia, Pennsylvania, USA

**Keywords:** aorto-enteric fistula, duodeno-caval fistula, entero-cutaneous fistula, entero-enteric fistula, gastro-cutaneous fistula, gastrointestinal fistula, peri-anal fistula, tracheoesophageal fistula

## Abstract

Gastrointestinal fistulas are increasingly being encountered in our clinical practice because of the increased burden of Crohn's disease, bariatric surgeries, interventional endoscopic procedures, nonsurgical trauma, and war and disaster zones worldwide. Presentation depends on the location and specific type of the fistula. Symptomatic ones can have a tremendous impact on social life and can cause dehydration, electrolyte imbalance, malnutrition, increased morbidity, and mortality. Different imaging studies and endoscopic procedures are done to establish the diagnosis. Treatment modalities to close the fistula depend on the underlying disease and the type of fistula. They include conservative treatment, medical therapy, endoscopic interventions, and surgery. Currently, there is no accepted treatment algorithm due to a lack of controlled clinical trials. The prognosis varies from fistula to fistula, and the mortality can be as high as 50%.

## 1. Introduction

A gastrointestinal (GI) fistula is an abnormal duct-like communication between the GI tract and another epithelial-lines surface such as the skin (external fistula) or another adjacent hollow organ or vascular system (internal fistula). Any part of the GI tract can be involved in fistulous communication. They are named after the organs connected by the fistula; the name of the organ of origin (active disease) comes first, followed by the name of the organ where the fistula ends (free of disease). GI contents (water, electrolytes, nutrients, and digestive fluid) leak from the GI tract into the connected organ through the fistula. The symptomatology depends on the organs involved, the underlying disease, and the fistula's size, site, acuity, and complexity. Postsurgical and Crohn's GI fistulas are commonly encountered in our clinical practice. Irrespective of the etiology, GI fistulas are associated with increased morbidity and mortality, and social life is significantly affected. With the advent of the lumen-apposing metal stent (LAMS) in 2011, endoscopic ultrasound (EUS)– guided different GI fistulae are made for palliative, therapeutic, and diagnostic purposes. Understanding GI fistulas is crucial for healthcare professionals because these conditions are associated with significant morbidity and mortality and unhappy social life, primarily due to complications such as sepsis, malnutrition, and fluid/electrolyte imbalances. Prompt recognition and appropriate management are essential to reduce mortality, prevent complications, and improve patient's quality of life. This review will discuss the etiopathogenesis, epidemiology, classification, clinical aspects, investigations, treatment, and prognosis of GI fistulas.

## 2. Brief Methods

Electronic data bases were searched to get help writing this review. These include *PubMed, Central* (PMC), *Google Scholar, Google, UpToDate and*openevidence.com*. The literature was* selected, screened, or synthesized searching the different studies and reviews of GI fistulas. Although some congenital GI fistulas were mentioned, most of the GI fistulas discussed in this review were adult GI fistulas. Otherwise there were no inclusion or exclusion criteria. The time period of the search was 2 months. There were no language restrictions. The literature search was done in English language.

### 2.1. Etiopathogenesis

GI fistulas most commonly occur as a postoperative complication of abdominal surgery (75%–85%) when the GI tract is manipulated [1]. Spontaneous causes of GI fistulas (15%–25%) include Crohn's disease, diverticular disease, malignancy, radiation treatment for malignancy, perforation of peptic ulcers, gallstone disease (Bouveret syndrome), appendicitis, ischemic bowel disease, *tuberculosis*, and actinomycosis. Gastro-cutaneous fistula (GCF) can occur after removal of long-term gastrostomy tube placement, iatrogenic injury (following dehiscence of gastroenteric anastomosis, following conventional gastric surgery, postbariatric surgery, splenectomy, and erosion from a drain), gunshot injury, perforated gastric ulcer, pancreatitis, pancreatic abscess, pancreatic cancer, and radiation injury. Nonsurgical injuries such as stab wounds and gunshot wounds can penetrate the abdominal wall and the gut, leading to GI fistulas. Road traffic accidents with injury to the intestine can also lead to GI fistulas [2]. Erosion of indwelling tubes can lead to GI fistulas as well [3]. Congenital enterocutaneous fistula (ECF) can occur due to complete failure of the omphalomesenteric duct to obliterate. Other congenital GI fistulas include tracheoesophageal fistula (TEF), esophageal-cutaneous fistula, rectourinary fistula, rectovaginal fistula, rectoperineal fistula, and perianal fistula (PAF) [4]. Development of GI fistula after surgery depends on many factors, which include infection, anastomotic leak due to wound dehiscence (as a result of inadequate blood supply, systemic hypotension, suture line tension, diseased bowel segments, and infection in the perianastomotic area), unrecognized bowel injury, the underlying disease, complexity of the surgery, surgeon's skill, improper surgical technique and incorrect placement of drainage [5]. ECF can occur following adhesion-lysis, incisional hernia repair, cancer surgery, inflammatory bowel disease (IBD) surgery, ileoanal anastomosis (J-pouch) surgery, drainage of intra-abdominal collection, small bowel resection, and Meckel's diverticulum resection [6]. Mucus fistula from the Hartmann pouch can occur after subtotal colectomy in patients with ulcerative colitis. Crohn's disease can cause transmural inflammation, deep ulcerations, and microabscess formation in the bowel wall. The ulcerated area may penetrate through the wall of an adjacent hollow organ or skin, leading to fistula formation. The exact pathogenesis of fistulizing Crohn's disease is poorly understood. The transformation of intestinal epithelial cells to mesenchymal-like cells is the initiating force. Increased concentrations of proinflammatory cytokines (TNF, IL-13, and TGFβ) can be found around the fistula [7]. Activation of matrix metalloproteinases (MMP-3 and MMP-9) also plays an important role in the development of Crohn's fistula. Several genetic mutations play an important role in the development of Crohn's fistula. The most consistently implicated gene is NOD2. Other genes involved in fistulizing Crohn's disease are immunity-related GTPase family M (IRGM) gene and NCF4 gene. Currently, genetic testing is not recommended for routine clinical use. The most commonly identified environmental risk factor is smoking which can impair mucosal immunity and alter gut microbiota (microbial dysbiosis, i.e., reduced diversity and increased proinflammatory bacteria) that may promote chronic inflammation and mucosal injury leading to fistula formation. Other environmental risk factors include diet high in saturated fat and animal protein, processed food, food additives, childhood antibiotics use, lack of breast feeding, urban living, and air pollution. Crohn's disease commonly causes PAF, ECF, enteroenteric fistula, enterocolonic fistula, enterovesical fistula, enterovaginal fistula, and rectovaginal fistula [8]. Colonic diverticulosis (more commonly left-sided or sigmoid diverticulosis) can cause colovesical fistula or colovaginal fistula in 4%–20% of patients due to inflammatory involvement of adjacent organ or as a result of surgery for complicated diverticulitis [9]. There are many case reports mentioning that colonic diverticulosis can also cause colocolonic fistula, coloenteric fistula, colouterine fistula, colosalpingeal fistula, colocutaneous fistula, and colocholecystic fistula [10–15]. Malignant fistula can occur when GI, pancreatic, and gynecological malignancies spread radially into the surrounding organs [16]. Radiation can cause chronic enteritis with poor healing, leading to GI fistulas (enterocutaneous, enterovaginal, and colovesical) after many months or years [17, 18]. In case of malignant biliary obstruction where endoscopic retrograde cholangiopancreatography (ERCP) is not feasible, biliary drainage is done by making a fistulous communication between the biliary system and the GI tract (choledochoduodenostomy or cholecystogastrostomy) placing an EUS-guided LAMS. EUS-guided gallbladder drainage is done by placing a covered self-expandable metal stent (SEMS) or LAMS between the gallbladder and stomach (cholecystogastrostomy) in patients with acute cholecystitis who are unfit for surgery. In benign or malignant gastric outlet obstruction (GOO), EUS-guided LAMS can be placed between the stomach and jejunum (gastrojejunostomy). In patients with Roux-en-Y gastric bypass, ERCP is challenging. In these cases, an EUS-guided transgastric fistula is made using a LAMS to facilitate peroral ERCP, also called an endoscopic ultrasound-directed transgastric ERCP (EDGE) procedure [19]. GI fistula can also involve the vascular system. The thoracic and abdominal aorta and inferior vena cava lie close to the GI tract. Although any part of the GI tract from the esophagus to the colon can be involved in aortoenteric fistula (AEF), the third part of duodenum is the most common site of involvement because of its fixed retroperitoneal location [20]. Aortic reconstructive surgery is the most common cause of AEF. Spontaneous rupture of atherosclerotic aortic aneurysm into the intestine is the most common cause of primary AEF. Other causes of primary AEF include septic aortitis, peptic ulcers disease, foreign bodies in the GI tract, biliary calculi, diverticulitis, carcinoma of the intestine, radiation and rarely, traumatic, mycotic, tubercular, or syphilitic aortic aneurysm ruptures into the intestine [21, 22]. Most of the time, the third or fourth part of the duodenum is involved followed by jejunum and ileum [23]. These are primary AEF without any prior vascular grafting. Secondary aortoenteric fistula (SAF) is more common than the primary AEF and is an important complication of endovascular or open abdominal aortic grafting. Generally, an infected or eroded aortofemoral or aortoiliac prosthetic grafting is involved. Usually, SAF occurs between the proximal end of the graft and the third part of the duodenum. The ileum is involved if the fistula occurs at the distal end of aortoiliac prosthetic grafting [24]. Duodeno-caval fistula (DCF) has been reported following penetrating abdominal injury, migration of inferior vena cava filter, transmural migration of ingested foreign body (chicken bone and toothpick), peptic ulcer disease, retroperitoneal tumor resection followed by radiotherapy and bevacizumab therapy [25, 26]. PAF or fistula-in-ano most commonly results from anorectal abscess, which develops as a result of infection and obstruction of anal glands. The abscess is located near the anal sphincter and finds its way into the perianal area with the formation of PAF. Incision and drainage of anorectal abscess can also develop PAF in one-third of cases [27]. Other causes of PAF include Crohn's disease, anal cancer, radiation proctitis, vaginal deliveries with episiotomy or third-degree or fourth-degree tears, sexually transmitted anorectal infections (gonorrhea, *chlamydia*, syphilis, and herpes simplex), ileal pouch-anal anastomosis (IPAA) due to anastomotic leak, perianal *tuberculosis* in endemic regions [28, 29], actinomycosis and hidradenitis suppurativa. Receptive anal intercourse can carry a higher risk for infections that can eventually lead to a fistula. Rarely, Nissen fundoplication (either open or laparoscopic) can cause esophagogastric fistula or ‘double lumen esophagus' [30]. TEF can be benign or malignant. Benign TEF can be congenital or acquired. Congenital TEF is associated with esophageal atresia in 87% of cases. Benign acquired TEF is usually secondary to prolonged use of endotracheal or tracheostomy tubes, esophageal stenting, radiation injury, foreign body ingestion (button battery and corrosive) [31, 32], or granulomatous disorders (*tuberculosis* and sarcoidosis) [33]. Malignant TEF generally occurs secondary to malignancy of the esophagus, lung, or tracheobronchial tree [34].

### 2.2. Epidemiology

The incidence and prevalence of GI fistulas vary from region to region and country to country. As mentioned, postoperative fistula is the most common cause of GI fistulas. But the rate of formation of fistula depends on the type of surgery: total gastrectomy—0%–28%, laparoscopic gastric bypass surgery—2%–5%, open gastric bypass surgery—2%–3%, sleeve gastrectomy—5%, esophagectomy—2%, pancreaticoduodenectomy—5%–8%, choledochoduodenostomy—5%–19%, elective colectomy—5%, and liver transplantation—2%–9% [35]. Crohn's disease is the most common cause of spontaneous fistula in developed countries. As many as 50% of patients with Crohn's disease can develop a fistula within 20 years of initial diagnosis [36]. One population-based databased analysis showed that the prevalent cases of fistulizing Crohn's disease in the United States in 2017 was ∼76,600, and out of them, the anal fistula was ∼52,900, rectovaginal ∼7400, enterocutaneous ∼2300, and internal ∼14,100. In the United States, at a given point, 11.7% of patients with Crohn's disease have GI fistula—8.1% anal, 1.1% rectovaginal, 0.3% enterocutaneous, and 2.2% internal [37]. Patients with Crohn's colitis and proctitis have a higher incidence of PAF. ECF is the most common type (88.2%) of all GI fistulas accounting for 89.1% of postabdominal surgery fistula [38, 39]. The incidence of Crohn's PAF and rectovaginal fistula is decreasing or stable, whereas the incidence of ECF and diverticular fistula remains stable without any evidence of decline. In Crohn's disease, ECF can occur in 12%–24% of patients after 10–20 years of diagnosis. About 1.5% of patients develop ECF after laparotomy for trauma [40]. In war and disaster zones, traumatic abdominal injuries are associated with a higher incidence of GI fistulas [41]. Rectovaginal fistula secondary to obstetric injury is higher in low-income and less-developed countries than in developed countries (0.2 per 1000 deliveries in Norway vs. 1.7 per 1000 deliveries in Southeast Asia and sub-Saharan Africa) [42]. PAF is a common anorectal disease. The prevalence is higher in men (12.3 cases per 100,000 population) than in women (5.6 cases per 100,000 population) [43]. Patients with colostomy or ileostomy can also develop peristomal ECF, that is, communication between the stoma and/or intestine and peristomal skin. The incidence of peristomal fistula following loop ileostomy is 0%–2.7% and following loop colostomy is 2.8%–5.1% [44]. Less than 5% of patients with acute diverticulitis of the colon can develop fistula, most commonly colovesical fistula in men and colovaginal fistula in women. Colonic fistula can also occur in about 20% of patients undergoing surgery for acute complicated diverticulitis [45]. About 14% of patients with radiation enteritis can develop radiation-induced fistula after a median latent period of 20 months [46]. A population-based birth defects data in the United States showed that the prevalence of congenital TEF is 2.3 per 10,000 live births and the incidence is about 1 in 3500 to 1 in 4500 live births [47]. About 5%–15% of esophageal cancer and 1% of tracheobronchial malignancy can develop TEF [48]. The incidence of primary AEF is around 0.007 per million annually, whereas that of SAF is about 1% (0.6%–2%) [49]. The incidence of DCF is extremely rare, and only a handful of documented cases have been reported in the literature [50].

Classification of GI fistulas is based on the anatomy, physiology, and etiology of the fistula as shown in Table 1. Anatomically, GI fistulas are divided into external and internal fistulas. External fistulas include ECF, GCF, and PAF. ECF connects the GI tract to the skin. A subset of the ECF is the enteroatmospheric fistula, the channel between the GI tract and the atmosphere. EAF is seen in patients with an open abdomen where loops of bowel are seen within the wound. This is generally iatrogenic following surgery for intra-abdominal hypertension, abdominal compartment syndrome, abdominal sepsis, or pancreatic necrosis [51]. Any part of the GI tract can be affected by ECF but the small intestine is the most common site of involvement followed by colon, stomach, and duodenum.

**Figure 3:**
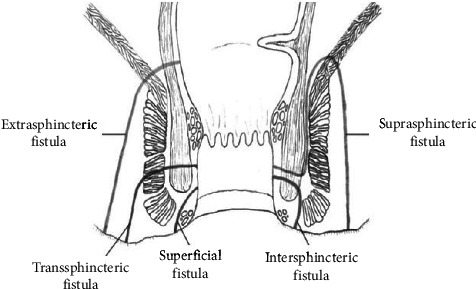
Anatomic classification of perianal fistula.

PAF is the most common external fistula seen in Crohn's disease. It is divided into four types as per the Parks classification (Figure 3): (1) Intersphincteric fistula (most common—50%–80% of all types)—fistulous track is confined to the intersphincteric space with its external opening near the anus; (2) transphincteric fistula—fistulous track passes through the external anal sphincter; (3) suprasphincteric fistula (transpuborectalis)—fistulous track crosses the internal anal sphincter, puborectalis muscle, ischiorectal fossa, and skin without involving the external anal sphincter; (4) extrasphincteric fistula (translevator ani—least common)—the fistulous track travels through the levator ani muscle above the dentate line and ischiorectal fossa to the skin [52]. A superficial perianal fistula extends from the lower part of the anal canal to the perianal skin without involving the sphincter or anal glands.

Another classification of perianal fistula is based on MRI imaging (St. James University Hospital Classification).

• Grade 1: simple intersphincteric fistula.
• Grade 2: intersphincteric fistula plus the presence of an abscess or another fistulous tract.
• Grade 3: transphincteric fistula.
• Grade 4: transphincteric fistula plus an abscess or another fistulous tract in the ischiorectal fossa.
• Grade 5: Translevator or supralevator fistula.

PAF is also classified into simple and complex PAF. Simple PAF has a single tract or subcutaneous tract or has involved < 30% of the external anal sphincter. Complex PAF has multiple tracts or has involved > 30% of the external anal sphincter, is due to Crohn's disease or radiation, or is recurrent.

The internal fistula communicates between the GI tract and another internal hollow organ. The common ones are (a) gut to gut: enteroenteric fistula, enterocolonic fistula, gastrocolonic fistula, duodenocolonic fistula, esophagogastric fistula, and pouch-pouch fistula and (b) gut to the adjacent hollow organ: enterovesical fistula, enterovaginal fistula, colovesical fistula, rectovaginal fistula, ileal pouch-vesical fistula, TEF and esophagobronchial fistula. Ileosigmoid fistula is the most common internal fistula seen in Crohn's disease. Another subtype of internal fistula is vascular-enteric fistula or enteric-vascular fistula, that is, vascular system to gut or gut to vascular system and the typical ones are AEF and DCF. It is essential to know the fistulous anatomy as it gives an idea about spontaneous closure of fistula and helps in planning operative closure. Physiologically, fistulas are graded according to the amount of output from the fistula. Fistulous output can predict metabolic consequences, morbidity, and mortality and help us correct the fluid, electrolytes, and nutritional deficiencies [53]. When the output from the fistula is less than 200 mL per day, it is called a low output fistula. A moderate output fistula is when the output is 200–500 mL/day, and a high output fistula is when the output is more than 500 mL/day. Etiologically, GI fistula can be classified as benign GI fistula and malignant GI fistula. Benign GI fistula include postsurgical fistula, Crohn's fistula, traumatic fistula, obstetric fistula, radiation-induced fistula, diverticular fistula, congenital fistula, and vascular-enteric or enterovascular fistula due to infected vascular prosthesis, aortic aneurysm, or miscellaneous causes. Malignant GI fistula occurs due to malignancy of the GI tract or adjacent organs.

### 2.3. Clinical Aspects

The symptomatology of GI fistula depends on the type of fistula. The main clinical finding of external fistula is the fistulous opening on the anterior or lateral abdominal wall or perianal area. Most of the time, ECF is iatrogenic, and patients present in the first week of postoperative period with abdominal pain, tenderness, wound infection with abscess formation, fever, leukocytosis, and ileus. ECF is observed after drainage of the abscess [54]. Leakage of GI content or feces to the skin causes excoriation and infection of the skin, leading to malnutrition, increased morbidity, and mortality. GCF has a high output, as more than 1 L of gastric juice is lost daily. Fluid and electrolyte loss may lead to dehydration, metabolic acidosis, and renal insufficiency. Patients with enteroenteric fistula are less symptomatic and do not require any surgical correction except treatment of the underlying disease [55]. Enterocolonic fistula is more symptomatic. Patients generally present with large-volume diarrhea [56]. Common symptoms of enterovesical fistula include pneumaturia, fecaluria, and recurrent urinary tract infection (UTI) [57]. A patient with an enterovaginal or rectovaginal fistula notices the passage of air, enteric fluid, or stool through the vagina [58]. PAF usually causes constant throbbing pain, smelly discharge (blood, pus, serous, or serosanguinous) from the perianal area, and recurrent perianal sepsis. Physical examination may reveal perianal skin irritation, redness, and swelling [59]. GI fistula decreases patient's quality of life profoundly affecting physical, psychological, and social domains. Pain, drainage, malodorous discharge, skin irritation, frequent need for wound care, dependence on medical devices (e.g., fistula appliances and seton), and healthcare professionals lead to restrictions in daily activities, social isolation, loss of independence, and increased psychological distress. Patients develop anxiety and depression. The impact on sexual function is pronounced, particularly in young women. Adults with TE fistula generally present with coughing after swallowing food [60]. They may have a prominent history of malignancy, radiation therapy, foreign body ingestion, prolonged endotracheal intubation, or granulomatous disorders. On the other hand, respiratory distress, choking, coughing, cyanosis, and feeding difficulty are the common symptoms of infants with congenital TE fistula. Most of the time, congenital TE fistula is associated with proximal esophageal atresia, and about one-third of patients with congenital TE fistula also suffer from congenital heart diseases, most commonly ventricular septal defect [61]. Patients with AEF classically present with intermittent minor GI bleeding (‘herald' or ‘sentinel' bleeding) followed by massive upper GI bleeding within a few hours to days. But in many cases, massive upper GI bleeding is the initial presentation [62]. The classic triad of abdominal pain, GI bleeding, and palpable abdominal mass is observed in only 6%–12% of cases in clinical practice [63]. A high index of suspicious is needed for timely diagnosis. DCF is another life-threatening condition of enterovascular fistula. Presentation is usually nonspecific, but about half the cases show signs of sepsis and GI bleeding. The septic features may vary from isolated fever to septic shock, and GI bleeding may range from occult GI blood loss to overt GI bleeding with hypovolemic shock [64].

### 2.4. Investigations

Usually, imaging studies are carried out to confirm the diagnosis of GI fistulas. Imaging studies not only define the exact anatomy of the fistula but also give an idea about the underlying disease processes and help in planning a successful treatment strategy. The particular imaging study varies from fistula to fistula. The most commonly used modality for assessing ECF is a CT scan with oral and intravenous contrast unless contraindication exists [65]. It may show the fistulous tract, any local abscess, and features of underlying disease processes such as IBD or malignancy. The fistulous tract is visible as a tubular structure (either collapsed or ‘tram-track' appearance due to gas or fluid content) originating from a loop of intestine, which passes through the peritoneum and the abdominal wall to reach the cutaneous surface. The sensitivity of detecting ECF by contrast-enhanced CT scan is about 85% [66]. MRI with oral and intravenous contrast is equally effective and can demonstrate the fistulous tract as a hyperintense fluid-filled tract (on T2 images), inflammatory versus stenotic disease, presence of abscess, and associated active disease [67]. In the absence of sepsis, fistulography is a valuable test to delineate the fistulous tract [68]. Small bowel follow-through (SBFT) is rarely used nowadays because of its decreased sensitivity in detecting ECF [69]. Transabdominal bowel/intestinal ultrasound is another noninvasive test mostly used in European countries to evaluate ECF. It is visualized as a hypoechoic peri-intestinal lesion in the region of intestinal wall thickening. It is highly operator-dependent but one study found it to be high sensitivity (87%) and specific (90%) in detecting ECF [70]. Hydrogen peroxide–enhanced ultrasound fistulography (after injecting hydrogen peroxide through the skin orifice) is also a highly sensitive test in which ECF can be visualized as a hypoechoic or anechoic duct-like structure between the intestine and the skin [71]. Endoscopic evaluation is often done to visualize the ECF and assess the underlying disease. By esophagogastroduodenoscopy (EGD), enteroscopy, and colonoscopy, the internal opening of the fistula can be visualized as an erythematous and slightly elevated area, but this can be missed easily because of the small size [72]. GC fistula can be confirmed by doing an upper endoscopy. Detection of the internal opening of ECF can be challenging, and sometimes, a soft-tip guide wire is probed, or methylene blue, betadine, or hydrogen peroxide is administered. CT scan, MRI examination, or conventional contrast-enhanced studies such as SBFT or barium enema (BE) can diagnose internal fistula. In one study, the sensitivity of detecting internal fistula by computerized tomography enterography (CTE) and magnetic resonance enterography (MRE) was much higher (sensitivity = 0.91) than conventional contrast-enhanced studies (sensitivity = 0.73), and there was no significant difference in the diagnostic efficacy between CTE and MRE [73]. Perianal fistula is detected by MRI, EUS, fistulography, examination under anesthesia (EUA), proctosigmoidoscopy, or probing the fistula with a long thin probe. A prospective study showed that dynamic contrast-enhanced MRI (DCEMRI) had very high sensitivity (97%) and specificity (100%) in the detection of perianal fistula [74]. MRI has now become the investigation of choice for the detection and classification of perianal fistula. In the case of a congenital TE fistula, water-soluble contrast is introduced into the esophagus under fluoroscopic guidance to visualize the fistula. Adult TE fistula is usually detected by bronchoscopy, esophagoscopy, or esophagogram with a water-soluble contrast agent. Bronchoscopy is most valuable in localizing the TE fistula. The diameter of the fistula, distance from the carina, and distance from the vocal cord should be documented. Simultaneous bronchoscopy and esophagoscopy with air or methylene blue instillation are more helpful in evaluating TE fistula. CT scan is complementary in assessing the extent of the TE fistula and the underlying disease in the esophagus, lungs, and mediastinum. Lateral chest X-ray may show a dilated esophagus distal to the fistula and evidence of pulmonary aspiration [75]. CT angiogram (CTA) is the investigation of choice in patients with AEF. It has high spatial resolution, rapid acquisition, and widespread availability. CTA findings include identification of aortic graft within the intestinal lumen, active extravasation into the intestinal lumen, loss of periaortic or perigraft fat plane, and the fat plane between the aorta and bowel wall, perigraft soft tissue, fluid or hematoma, ectopic gas, and aneurysm or pseudoaneurysm [76]. MRI has superior soft tissue contrast and avoids ionizing radiation. But it has lower spatial resolution than CT, longer acquisition times, and is limited by artifacts from metallic implants. It is not suitable for unstable patients and is less available in emergency situation. In a hemodynamically stable patient, upper endoscopy should be done in an appropriate setting to visualize the actual erosion into the duodenal wall of third or fourth part of the duodenum. However, the sensitivity of diagnosing AEF by upper endoscopy is about 50% [77]. Similarly, the presence of DCF can be confirmed by a CT scan, CTA, MRI of the abdomen, or cavography. These may demonstrate fistulous communication between the duodenum and inferior vena cava or thrombus with intraluminal air in the inferior vena cava suggestive of DCF [25, 64, 78]. Upper endoscopy may reveal bleeding, a blood clot, duodenal ulcer, or a penetrating foreign body in the duodenum [79]. During endoscopy, large amount of air can enter the inferior vena cava via the fistula leading to air embolism (pulmonary embolism or systemic embolism). A case of fatal cerebral embolism was reported after upper endoscopy in a patient with DCF [80]. Routine diagnostic upper endoscopy is not recommended in patients with DCF.

## 3. Treatment and Prognosis of GI Fistulas

### 3.1. External Fistulas

#### 3.1.1. ECF

**Figure 4:**
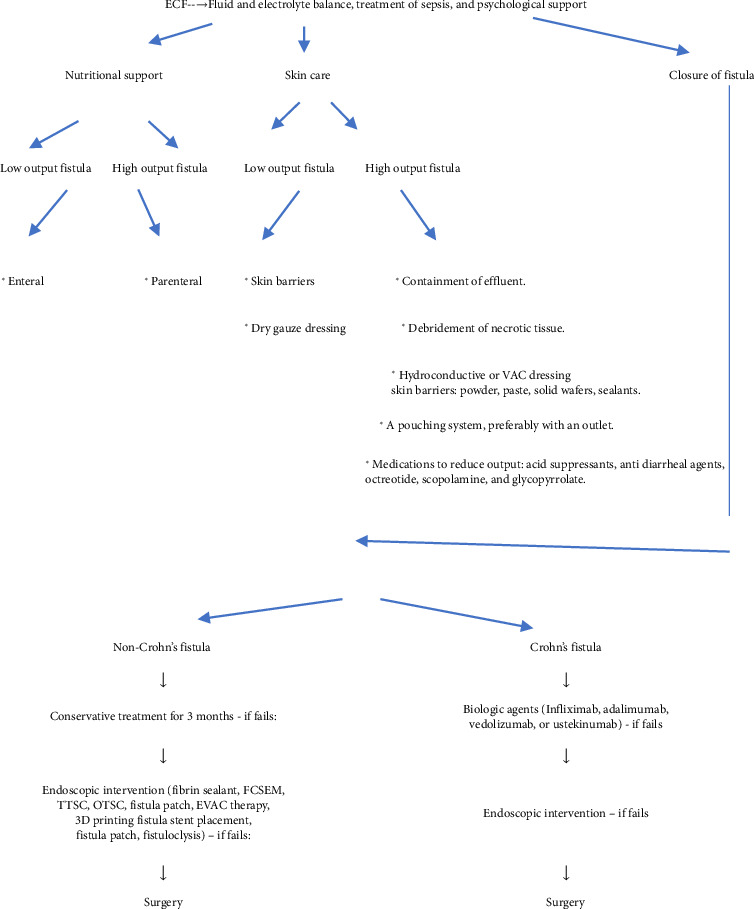
Summary of treatment of ECF.

A multidisciplinary approach is required to manage ECF. Fluid and electrolyte balance, treatment of sepsis, nutritional support, medical management of fistula output, wound/skin care, fistula closure, and psychological support are the main steps of management. As patients with ECF can lose large amounts of fluid and electrolytes, particularly in high output fistula, the primary aim will be to replace these lost fluid and electrolytes by giving crystalloids parenterally [81]. Patients with high output fistula will also require urinary catheterization to maintain fluid and electrolyte balance. Most of the patients with ECF have underlying intra-abdominal sepsis, which should be treated with appropriate antibiotics and drainage of the abscess (CT or ultrasound-guided). Nutritional support is essential to decrease the morbidity and mortality [82]. There is an increased demand for nutrition (calories, protein, vitamin C, and trace elements such as zinc, copper, and selenium) in patients with ECF because of continuous loss of nutrition through the fistula, increased catabolism due to sepsis, and inadequate intake of calories [83]. One study showed that patients with ECF taking at least 1500 kcal/day had a 3.6-fold decrease in mortality compared to those whose calorie intake was less than 1500 kcal/day [82]. Most patients with ECF require enteral and parental nutrition except a subset of patients with distal GI tract fistula or low output fistula, which can be managed by giving only enteral nutrition. The length of the intestine from the ligament of Treitz to the external fistula should be at least 4 feet for enteral nutrition to be successful. High-calorie drinks, polymeric formulas, semielemental nutrition, or immunomodulated nutrition can be given for enteral nutrition. Total parenteral nutrition is typically required in patients with high output fistula, short bowel length (< 75 cm), intestinal discontinuity, failure to get enteral feeding access, symptomatic intolerance to enteral feeding, and worsening of fistulous output after enteral feeding [84]. Different medications have been tried to decrease the high output fistula. These include acid suppressants (proton pump inhibitors and H2 blockers), antidiarrheal agents (loperamide, diphenoxylate/atropine, tincture of opium, and codeine), scopolamine, glycopyrrolate, and octreotide. They have positive result in reducing the fistulous output. Octreotide should be tried in high output fistula and should be continued if there is significant improvement. Some studies suggest that the administration of octreotide can help in the spontaneous closure of the fistula [85]. Wound care and skin care are the vital steps in managing ECF. Effluent should be contained to promote the healing of the wound and prevent the breakdown of the skin. Debridement of any necrotic tissue in the wound should be done to allow granulation tissue to form. Hydroconductive or vacuum-assisted closure (VAC) dressings can be applied to promote perifistular wound healing. The perifistular skin should be kept dry and clean. Skin barriers such as powder, paste, solid wafers, or sealants should be used to prevent contact of the effluents to the skin. In case of low output fistula, skin barriers, dry gauze dressings, and dressing a few times a day should be enough to contain the effluents. In the case of a high-output fistula, a pouching system, preferably with an outlet system, should be used for better drainage and emptying [86]. Closure of the fistula depends on the underlying cause. Crohn's associated ECF responds better to medical therapy than non-Crohn's associated ECF. Several medications have been tried to close Crohn's ECF. These include 6-marcaptopurine (6-MP), azathioprine (AZA), methotrexate (MTX), and biologic agents. 6-MP or AZA effectively closes Crohn's fistula in 30%–40% of cases [87]. One small study showed that combining infliximab with long-term 6-MP or AZA caused the complete closure of the fistula in 75% of cases; the median time of complete closure of the fistula was 2 weeks, and the fistula remained completely closed for more than 6 months [88]. One small case series found MTX to be effective in closing Crohn's fistula in 25% of cases [89]. Another study revealed that 55% of patients receiving infliximab had closure of Crohn's fistula [90]. Infliximab is now considered as the first line biologic agent in the induction and maintenance of Crohn's fistula healing. Other biologic agents such as adalimumab, vedolizumab, and ustekinumab were also found to have efficacy in closing Crohn's fistula [91, 92]. Vedolizumab and ustekinumab are now used as second-line biologic agents in patients who had inadequate or failed response to anti-TNF therapy. A small study found that despite the initial success of the biologic agents in closing Crohn's ECF, more than 50% of patients ultimately required surgical intervention to close the fistula [93]. In the case of non-Crohn's fistula, closure of the fistula is considered if the ECF remains patent despite 3 months of conservative treatment (fluid and electrolyte balance, control of sepsis, nutritional support, skin, and wound care). Definitive surgical closure of the fistula is usually performed when normal physiology is restored and the patient is stable without any sepsis [94]. The diseased intestinal segment is resected with possible primary anastomosis. All the unhealthy tissues of the fistulous tract are debrided, and then the fistula is closed with the viable and healthy tissue edges. Surgical success in closing ECF is 80%–95%, but the recurrence rate can be as high as 14%–36% [95, 96]. Resection with hand-sewn anastomosis has lower recurrence (11%) than stapled anastomosis (35%) and simple oversewing (22%–36%). Based on a recent population-based study, the risk ratio is approximately 6 and the odds ratio is approximately 190 for ECF closure with surgery compared to conservative treatment [97]. The recurrence rate can be lowered if the involved bowel is resected instead of oversewed or repaired [98]. A retrospective analysis showed that staged surgery without primary anastomosis of the ECF could increase the healing from 73% to 94% and decrease mortality from 12% to 6% [99]. Endoscopic intervention should be considered prior to surgical management. In one study, endoscopically delivered fibrin sealant (biodegradable fibrin glue) was highly successful in closing the postoperative ECF resistant to conservative treatment [100]. In another study, fibrin sealant was introduced through the external opening. It closed the ECF early and significantly decreased nutritional support and morbidity [101]. In case of high output ECF, fully covered self-expanding metal stents (FCSEMS) can be placed endoscopically at the site of ECF to divert the enteric effluents before doing definitive surgery for closure of ECF. Diversion of the effluents will allow cutaneous wound healing, early enteral feeding, improvement of nutritional status, short hospital stay, and decreased in-hospital mortality. Other methods of temporary diversion of enteric effluents through the ECF include endoscopic clip placement—through-the-scope clip (TTSC) or over-the-scope clip (OTSC), three-dimensional (3D) printing fistula stent placement, fistula patch, endosponge or endoscopic vacuum-assisted (EVAC) therapy, and fistuloclysis that is a technique of using fistula as the primary enteral port [102, 103]. Complete resolution of ECF can be achieved in 80%–95% of cases if sufficient waiting time, proper nutrition, and appropriate medical, endoscopic, and surgical treatments are given [54]. Spontaneous closure of ECF is higher if the enteric wall defect is small and noninfected, the fistula has a long tract, and the fistula arises from the esophagus, duodenal stump after gastric resection, jejunum, or pancreaticobiliary tract. Surgical correction is more likely to require if the fistula originates from the lateral wall of duodenum, duodenojejunal flexure, or ileum [104]. A flowchart summarizing the treatment algorithm is shown in Figure 4.

Measures to prevent postoperative fistulas include patient optimization, meticulous surgical technique with appropriate procedure selection, selective use of antibiotics, prevention of surgical site infection, and vigilant postoperative monitoring. For Crohn's fistula, a multidisciplinary approach is essential to optimize the outcome. Coordinated involvement of gastroenterologists, colorectal surgeons, radiologists, nutritionists, and specialized nursing staff is essential. Early and ongoing collaboration is critical for diagnostic workup, individualized treatment, and long-term follow-up.

#### 3.1.2. GCF

The fistulous tract is generally epithelialized, which should be cauterized electrochemically by introducing silver nitrate through the external (cutaneous) opening and applying a gold probe (BICAP) through the internal (gastric) opening [105]. Then, the fistula can be closed endoscopically by deploying endoscopic clips. Patients should be placed on intravenous proton pump inhibitors to increase gastric pH, somatostatin to decrease gastric secretion, and intravenous metoclopramide to improve gastric emptying. The patient should be placed on total parenteral nutrition. Hydration and electrolyte balance should be maintained. Any local or systemic infection should be treated with antibiotics. There is a high success rate in closing the GC fistula (< 1 cm) by the OTSC [106, 107]. There are two types of OTSC—the bear trap-shaped OTSC (OVESCO AG, Germany) and the flat, star-shaped OTSC (Padlock clip, Aponos Medical, USA)). In the case of OVESCO, gastric closure bear claw type is used, the tissue around the fistula is grasped and sucked into the cup with the scope channel suction, and then the ‘bear claw' clip is deployed [108]. The Padlock clip is deployed after suctioning the fistulous opening into the cup attached to the end of the scope. The six prongs lift the tissue and provide circumferential tissue approximation [109]. Upper GI series with gastrografin and upper endoscopy can be done 48 h after OTSC placement to confirm closure of the GC fistula. Other endoscopic methods of closing GC fistula include application of human fibrin sealant, and endoscopic stent placement particularly after sleeve gastrectomy and duodenal switch [110]. Large sized GCF (> 2 cm) can be closed by endoscopic suturing using an OverStitch device by Apollo Endosurgery [111]. Endoscopic intervention is now considered as the first-line treatment for the closure of GC fistula. Surgical closure of the fistula should be done if the electrochemical cauterization and endoscopic management fail to close the GCF [112]. The GC fistula can close spontaneously in about 6% of cases. The mortality from GC fistula ranges from 11% to 44% and occurs from electrolyte imbalance, malnutrition, and sepsis [113].

#### 3.1.3. Perianal Fistula

The treatment of perianal fistula depends on the underlying etiology and type of fistula. In non-Crohn's fistula, the underlying infection should be treated, and associated perianal abscess (Grade 2 and Grade 4) should be drained. The various modalities of closing the perianal fistula include fistulotomy, seton (a thin silicon string) placement, endorectal advancement flap (EAF) procedure involving mobilization of a partial thickness flap (rectal mucosa, submucosa, and some muscle fibers), ligation of intersphincteric fistula tract (LIFT), injecting fibrin glue, or bioprosthetic plug into the fistula under general anesthesia and reconstructive surgery. Fistulotomy is the treatment of choice for intersphincteric fistula (Grade 1) as it does not involve the external anal sphincter, and there is less chance of anal incontinence [59]. Transphincteric fistula (Grade 3) is usually treated by initial placement of a seton followed by fistulotomy. Seton placement can lower the fistulous tract making the external anal sphincter less involved so that fistulotomy can cause less chance of anal incontinence. Another treatment modality of transphincteric fistula is to dissect the intersphincteric groove, encircle, ligate and divide the transphincteric tract, and debride the external opening (modified LIFT technique) [114]. Suprasphincteric fistula can be managed by seton implantation followed by fistulotomy. Treatment of extrasphincteric fistula (Grade 5) includes EAF procedure, filling the fistula with fibrin sealant, or fistulotomy with reconstructive surgery [115]. Perianal fistula after IPAA is a complex fistula from anastomotic defect and can be treated by EAF, gracilis muscle flap, Martius flap (bulbocavernosus), sealant, seton, or fistulotomy with a success rate of 64% [116]. Managing Crohn's perianal fistula requires a multidisciplinary team of gastroenterologists, radiologists, and surgeons. Treatment is offered to symptomatic patients who do not have active proctitis. Pharmacologic therapy is tried first to close the fistula. If the pharmacologic therapy fails, surgical intervention is required as mentioned above. Initial treatment with antibiotics (metronidazole 750–1500 mg/day or ciprofloxacin 1 gm/day for 10 weeks) has shown to improve symptoms, temporarily treating local infections and closing the fistula. One small study showed response and remission occurred in 30% of patients treated with ciprofloxacin [117]. As shown in open-label studies, metronidazole can close the perianal fistula in 34%–50% of patients [118]. However, relapse of the fistula frequently occurs after cessation of antibiotic therapy. Other treatment modalities for closing Crohn's perianal fistula include immunomodulator and biologic therapy. Fistula closure varies among different immunomodulators. In a randomized controlled study, 31% of patients treated with 6-mercaptopurine or azathiopurine had closure of Crohn's fistula [119]. In ACCENT II (A Crohn's Disease Clinical Trial Evaluating Infliximab in a New Long-Term Treatment Regimen in Patients with Fistulizing Crohn's Disease) trial, 46% of patients receiving maintenance dose of infliximab for 54 weeks had a fistula response [120]. The relapse of fistula was high (about 66%) after stopping the infliximab for a year. In the Crohn's trial of the fully Human Antibody Adalimumab for Remission Maintenance (CHARM) study, 33% of patients treated with adalimumab had complete closure of fistula [121]. In a study at St. Marks Hospital, adalimumab was administered to fistulizing perianal Crohn's disease patients who lost response or were intolerant to infliximab therapy. Forty-three percent of adalimumab-treated patients had a clinical response, and 14% had clinical remission at 6 months [122]. Different studies suggest that higher serum trough levels of anti-TNF drugs have been associated with higher rates of response or closure of the perianal fistula [123]. American College of Gastroenterology Clinical Guideline in 2018 recommended the addition of antibiotics to infliximab in treating perianal fistula [124]. When the perianal fistula is refractory to anti–tumor necrosis factor (anti-TNF) therapy, other medications can be tried. Tacrolimus can induce apoptosis of macrophages and inhibit the production of IL-12/IL-23P40 and TNF-α [125]. In one study carried out by Sanborn et al., oral tacrolimus 0.2 mg/kg body weight was found to improve perianal fistula but not complete remission [126]. A systematic review showed that ustekinumab (IL-12/IL-23 inhibitor) treated for active Crohn's perianal fistula for 12 months had a clinical response with at least a 50% decrease in fistula drainage in 53.9% of cases without achieving fistula remission [127]. In a meta-analysis, vedolizumab could heal the perianal fistula in about one-third of cases when administered to patients with previously failed anti-TNF therapy [128]. In a post hoc subanalysis of a global Phase 3 trial, risankizumab closed Crohn's fistula in 28.6% of the induction group and 57.1% of the maintenance group [129]. Small molecule agents have also been found to be useful in the treatment of Crohn's perianal fistula. In Phase 3 induction and maintenance trials, higher proportion of patients had complete resolution of perianal fistula and more than 50% reduction of drainage of perianal fistula in the upadacitinib (UPA) group than placebo group [130]. Crohn's perianal fistula refractory to the above therapy can be offered mesenchymal stem cell (MSC) therapy. MSC therapy is a new treatment modality, and few studies have been carried out on its efficacy in healing perianal fistula. One meta-analysis found that MSC therapy could cure Crohn's perianal fistula in 61.75% of cases [131]. MSCs (either allogenic or autologous) are isolated from adipose tissue, bone marrow, or an umbilical cord. They can differentiate into myocytes, adipocytes, chondrocytes, and osteocytes. They are essential in healing perianal fistula because of their anti-inflammatory, immunomodulatory effects, angiogenesis, and mitogenic properties [132]. The method of administration of MSC therapy includes (1) the location and type of the fistula is first thoroughly investigated by MRI and EUS to assess the volume of MSCs to be administered—5 × 10^6^ MSCs per mL most commonly used; (2) antibiotics should be given if there is any active infection, and incision and drainage should be done if there is any abscess greater than 2 cm; (3) pudendal nerve block is recommended for anesthesia as local anesthetic such as lidocaine can be harmful to MSCs; (4) antiseptics such as chlorhexidine, octenidine, or just normal saline should be used instead of povidone, alcohol, or hydrogen peroxide which could be damaging to MSCs; (5) next the fistulous tract should be curetted and irrigated with normal saline; (6) internal orifice of the fistula is then closed with an absorbable suture; (7) finally MSCs are delivered into the fistulous tract either by direct injection or in combination with fibrin glue or a fistula plug. The perianal fistula is evaluated for closure after 6, 12, and 24 weeks [133].

The prognosis of perianal fistula depends on the type and etiology of the fistula. Generally, non-Crohn's simple intersphincteric fistula heals up by 12 weeks if it is treated by fistulotomy. About 70%–80% of Crohn's perianal fistulas are complex, and their management is highly challenging because of their refractoriness to multimodal treatment strategies and recurrence after healing. Many surgical interventions are done in most of the patients. Many patients develop opportunistic infections from immunosuppressive medications, and many patients develop fecal incontinence from surgery. About 40% of patients continue to have active disease, and 10%–20% of Crohn's perianal fistula ultimately need proctectomy or proctocolectomy [134].

### 3.2. Internal Fistulas

#### 3.2.1. Gut to Gut Fistulas and Gut to Adjacent Hollow Organ Fistulas

No treatment is necessary for asymptomatic internal fistulas such as ileoileal or ileocecal fistulas [135]. Treatment of symptomatic patients is surgery but depends on the underlying etiology and type of fistula. Sometime, surgery has to be staged depending on patient's underlying medical condition, imaging studies, and intraoperative findings. Symptomatic improvement, fistulotomy with repair of gut continuity, and restoration of function of the other involved organs are the main aims of surgery. Crohn's symptomatic fistulas are first treated with medications that have been reported to close the fistula. These include infliximab, adalimumab, certolizumab, vedolizumab, ustekinumab, risankizumab 6-mercaptopurine, azathiopurine, mycophenolate mofetil, tacrolimus, and cyclosporine A [136, 137]. Surgical intervention is necessary if the medical therapy fails. A retrospective analysis of data from a large clinical trial found that infliximab-treated patients had cumulative healing rate of internal (enteroenteric, enterocolonic, gastroenteric, and gastrocolonic) fistulizing Crohn's disease as per MRI imaging was 15.4%, 32.3%, and 43.9% at 1, 3, and 5 years, respectively. After a median follow-up of 3.5 years, major surgery was necessary in 43% of patients [138]. A systematic review revealed that Crohn's enterovesical fistula responded to infliximab therapy with complete fistula closure in 57.1% of cases and partial fistula closure in 35.7% of cases. Rectovaginal fistula responded to anti-TNF therapy with complete healing of the fistula in 41% of cases and partial healing of the fistula in 21.8% of cases [139].

#### 3.2.2. TE Fistulas (TEF)

Surgical correction should be planned as soon as congenital TE fistula is diagnosed. Acquired TE fistula is initially treated by endoscopic intervention. If the fistula is located in the middle third or lower third of the esophagus, closure of the fistula is done by placing an esophageal fully covered or partially covered SEMS. In all other locations, airway stenting is done by doing bronchoscopy. Sometimes, double (combined airway and esophageal) stenting may be required to cover a large (> 2 cm) TE fistula or if there is tracheal stenosis or esophageal stent causes tracheal compression. In case of TE fistula induced by esophageal stenting, airway stenting is done first followed by placement of a new esophageal stent. The stents should cover areas 2 cm proximal and 2 cm distal to the fistula and be secured with endoscopic suturing or endoclips to prevent migration [140]. The clinical success rate of SEMS in treating TE fistula is 67%–100% [141]. Stent migration can be as high as 40%. Besides, SEMS can cause dysphagia, chest pain, gastroesophageal reflux, pneumonia, cough, nausea, bleeding, perforation, and tracheal compression [142]. In case of benign TE fistulas, definitive surgical treatment is considered when the patient's clinical status improves. The surgical treatment includes separation of esophagus and trachea, closure of the fistula with a muscle flap or omentum, esophageal bypass surgery, or resection of the lesion [143]. Patients with malignant TE fistula are malnourished and are on chemoradiation most of the time. SEMS are placed as a palliative measure to improve patient's quality of life. Alternative to SEMS, tissue adhesives such as cyanoacrylates, fibrin glue, and thrombin have been used for small TE fistula with variable success in different case reports although there is no controlled trial [144, 145].

#### 3.2.3. Vascular-Enteric Fistula or Enteric-Vascular Fistula

##### 3.2.3.1. AEF

AEF is a life-threatening condition needing emergent surgical intervention. Aggressive hemodynamic support should be given to resuscitate the patient. Blood culture should be done and broad-spectrum antibiotics should be started. Prompt surgical intervention is done by laparotomy, bleeding is controlled, the infected tissues are debrided, the fistulous opening of bowel wall is repaired, the involved aneurysm or infected aortic graft is resected, and then revascularization is done by closing the aortic fistulous opening either by stitching together the edges or placing a new graft [62]. The other method is endovascular repair of the aorta (EVAR) which involves placement of an aortic stent graft under fluoroscopic guidance via bilateral femoral artery cutdowns. Open surgical intervention is associated with higher (33.9% vs. 7%) in-hospital mortality but lower (19% vs. 42%) rate of sepsis when compared to EVAR [146, 147]. Open surgical repair is a high-risk surgery, but the nidus of infection is removed by debridement of infected tissue during surgery. EVAR is a minimally invasive procedure, and the new graft is placed in the contaminated field that leads to chronic infection, sepsis, and long-term use of antibiotics. There is no difference in long-term survival between these groups. So open surgery should be considered for SAF, and EVAR should be considered for primary AEF or even SAF in very sick patients as a bridge therapy with the plan of doing definitive open surgery at a later date. As a whole, mortality from AEF is almost 100% if untreated but nearly 50% within 60 days if treated surgically [148, 149].

##### 3.2.3.2. DCF

DCF is another life-threatening GI bleeding condition where emergent surgical intervention is needed. Patients should be given enough transfusion of blood and infusion of fluid to improve hemodynamic status. During on-gong resuscitation, surgical intervention should be done. This will include division of the fistula, repair of the inferior vena cava, and the involved duodenum. Usually, a diverting surgery such as gastrojejunostomy is done [26]. Other option is placement of an endovascular stent into the inferior vena cava as a temporary measure to stop the bleeding with an intention of doing definitive surgery on a later date when the patient is more stable [150]. Intravenous broad-spectrum antibiotics are started preoperatively and continued postoperatively for about a month followed by oral antibiotics for about 2 months. Antibiotics are modified depending on the culture report. Parenteral nutrition is given when enteral nutrition is on hold. The overall mortality of DCF can be as high as 50%–60% even after surgery for every etiology except in case of migrating vena caval filters (mortality 10%) and penetrating abdominal injury where the prognosis is slightly better [64]. The diagnostic tests and management strategies of various GI fistulas are shown in Table 2, and a summary of key studies of GI fistulas is shown in Table 3.

### 3.3. Limitations of the Evidence Regarding the Treatment of GI Fistulas

There is paucity of robust randomized controlled clinical trials for the treatment of GIF. Most of the data available are obtained from small heterogenous studies, case series, retrospective reviews, and expert opinion, specially for internal fistulas and non-Crohn's-related fistulas. There is no standardized, evidence-based algorithm for the endoscopic and surgical interventions of GIF. Much of the evidence for the pharmacologic management of Crohn's GIF is of low quality with significant reliance on post hoc analyses rather than primary end points in clinical trials. As a result, it is difficult to develop universally acceptable treatment recommendations and come to a firm conclusion.

Long-term follow-up of GI fistulas is essential because patients remain at risk of recurrence, ongoing complications (electrolyte disturbance, sepsis, and malnutrition), morbidity, and mortality despite closure of the fistula spontaneously or surgically. Mortality is higher in patients who do not have fistula closure with 1, 3, and 5 years mortality of 33.7%, 42.1%, and 47.6%. Long-term follow-up allows early detection of recurrence, nutritional support, and complications related to the fistula [97].

## 4. Summary

Out of various iatrogenic and noniatrogenic causes of GI fistulas, postoperative and Crohn's fistulas are most commonly encountered in our clinical practice. GI fistulas are classified into external and internal fistulas and low, medium, and high output fistulas. The symptomatology depends on the underlying cause, location, and type of the fistula. ECF is associated with intra-abdominal infection and can cause fluid and electrolyte imbalance, malnutrition, skin excoriation, and infection. Patients with perianal fistula presents with perianal pain, discharge, and recurrent perianal infection. Patients with internal fistulas have unique symptoms due to involvement of colon (diarrhea) or adjacent organs such as urinary bladder or vagina (passage of air, fluid, or stool per urethra or vagina). Congenital TE fistula is suspected in the neonatal period when the infant presents with respiratory distress or feeding difficulty. Coughing after swallowing food is the usual presentation of adult TE fistula in appropriate clinical scenario. Herald bleeding followed by massive GI bleeding is the usual presentation of AEF. But patients generally present with septic features with occult to massive GI bleed in case of DCF. Diagnosis of GI fistulas is confirmed by imaging studies (CT, CTA MRI, barium studies, water soluble contrast studies, and fistulography) and endoscopic procedures (EGD, colonoscopy, enteroscopy, bronchoscopy, and EUS). Management includes fluid and electrolyte balance, nutritional support, infection control, and closure of the fistula. In the case of ECF, pharmacological therapy is given to reduce the amount of effluent, and appropriate care should be taken to heal the wound/skin infection. The effluent can be diverted by placing a FCSEMS. Small ECF can be closed by putting fibrin glue or fibrin sealant. If the conservative measures fail, definitive surgery is usually done after few months when the patient is stable without any sign of infection. Medical therapy is tried first in patients with Crohn's ECF. Definitive surgery is considered after failure of medical treatment. Small GCF is first cauterized electrochemically, and then closed by OTSC. Large (> 2 cm) GCF will need endoscopic suturing or surgery. Non-Crohn's perianal fistula is treated by different surgical modalities depending on the type of the fistula. Crohn's perianal fistula is first treated by medical therapy, and if that fails, surgery is performed. MSC therapy is still under investigation to treat perianal fistula refractory to medical treatment. Similar to external fistulas, symptomatic gut to gut or gut to adjacent organ non-Crohn's internal fistulas are managed by surgery. Crohn's patients are first treated with medical therapy, followed by surgery if medical treatment fails. Congenital TE fistula is treated by surgery as early as possible. Acquired TE fistula is initially managed by stenting, followed by definitive surgery at a later date. AEF and DCV can present with life-threatening GI bleed, and both may require prompt resuscitation with massive replacement of blood and fluid, administration of intravenous antibiotics, and emergent surgical intervention or endovascular stenting. Mortality remains high despite aggressive management of these conditions. The controversy in the current literature regarding GI fistula primarily revolves around the optimal management strategies, particularly the balance between medical and surgical interventions, and the role of emerging endoscopic techniques. One significant area of debate is the management of fistulas in Crohn's disease. The AGA guidelines highlight the use of anti-TNF therapies as a mainstay of medical treatment, but the timing and indications for surgical intervention remain uncertain due to limited and low-quality data. The ACG also emphasizes the complexity of managing high output fistulas, which often necessitate surgical intervention, while low output fistulas may be managed with medical therapy alone or in combination with antibiotics and biologics. Another contentious issue is the role of endoscopic management. Endoscopic techniques, such as stent placement, clip closure, and EVAC, have shown promise in managing GI leaks and fistulas. However, there is no standardized, evidence-based algorithm for their use, and the choice of technique often depends on local expertise and device availability. The lack of large-scale, randomized controlled trials further complicates the establishment of definitive guidelines.

Future directions in the management of GI fistulas will be focused on a multidisciplinary approach including personalized wound care, utilizing advanced endoscopic techniques (closure of the fistula by TTSC, OTSC, stents, endoscopic suturing, fibrin sealants, and EVAC), minimally invasive surgery, and targeted biologic/MSC therapy in case Crohn's fistulas. Ongoing research will continue to establish optimal algorithms.

## Figures and Tables

**Figure 1:**
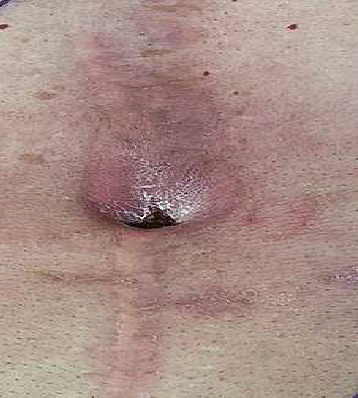
Cutaneous side of ECF.

**Figure 2:**
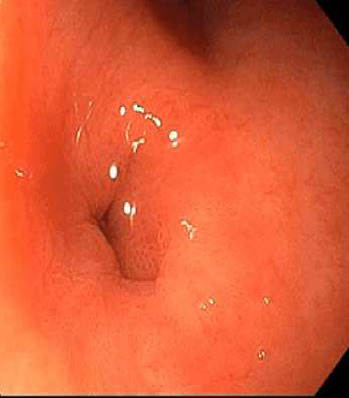
Gastric side of GCF.

**Table 1:** Classification of GI fistula.

Anatomical	External: ECF (Figure 1), GCF (Figure 2), and PAF
	Internal: (a) gut to gut: enteroenteric fistula, enterocolonic fistula, gastrocolonic fistula, duodenocolonic fistula, esophagogastric fistula, and pouch-pouch fistula
	(b) gut to the adjacent hollow organ: enterovesical fistula, enterovaginal fistula, colovesical fistula, rectovaginal fistula, ileal pouch-vesical fistula, TEF, and esophagobronchial fistula
	(c) vascular-enteric fistula: AEF and DCF

Physiological	(a) Low-output fistula: When the output from the fistula is less than 200 mL per day
	(b) Moderate output fistula: When the output is 200–500 mL/day
	(c) High output fistula: When the output is more than 500 mL/day

Etiological	(a) Benign GI fistula: postsurgical fistula, Crohn's fistula, traumatic fistula, obstetric fistula, radiations-induced fistula, diverticular fistula, congenital fistula, and vascular-enteric or enterovascular fistula due to infected vascular prosthesis, aortic aneurysm, or miscellaneous causes
	(b) Malignant GI fistula: adenocarcinoma, lymphoma, and squamous cell carcinoma

**Table 2:** Diagnostic tests and management strategies of various GI fistulas.

	Diagnostic tests	Management strategies
ECF	CT or MRI with oral and intravenous contrast. Transabdominal intestinal ultrasound	Multidisciplinary approach: fluid and electrolyte balance, treatment of sepsis, nutritional support, medical management of fistula output, wound/skin care, fistula closure, and psychological support
	Endoscopy, CTE, or MRE	

GCF	Upper endoscopy	TPN, antibiotics in case of local systemic infection. Electrochemical cauterization of GCF by silver nitrate and BICAP. Closure of the fistula by OTSC or endoscopic suture. Surgical closure if endoscopic closure fails

PAF	Dynamic contrast-enhanced MRI, EUS, fistulography, EUA, proctosigmoidoscopy, or probing the fistula with a long thin probe	In non-Crohn's PAF, the underlying infection should be treated, and associated perianal abscess (grade 2 and grade 4) should be drained. The various modalities of closing the perianal fistula include fistulotomy, seton (a thin silicon string) placement, endorectal advancement flap (EAF) procedure involving mobilization of a partial thickness flap (rectal mucosa, submucosa, and some muscle fibers), ligation of intersphincteric fistula tract (LIFT), injecting fibrin glue or bioprosthetic plug into the fistula under general anesthesia, and reconstructive surgery
		In Crohn's PAF, initial treatment includes antibiotics plus biologics. If that fails, surgical intervention or MSC therapy

Internal fistula	CTE, MRE, CT, MRI, SBFT, BE	Asymptomatic—no treatment
		Symptomatic non-Crohn's internal fistula—surgery
		Symptomatic Crohn's internal fistula—anti-TNF therapy. If that fails—surgery

TEF congenital	Esophagogram with a water-soluble contrast agent	Surgical correction as soon as possible

TEF acquired	Bronchoscopy, esophagoscopy, esophagogram with a water-soluble contrast agent or CT scan	Fistula located in the middle third or lower third of the esophagus, closure of the fistula is done by placing an esophageal fully covered or partially covered self-expanding metal stent (SEMS). In all other locations, airway stenting is done by doing bronchoscopy. Sometimes, double (combined airway and esophageal) stenting may be required to cover a large (> 2 cm) TEF or if there is tracheal stenosis or esophageal stent causes tracheal compression

AEF	CTA—investigation of choice	Aggressive hemodynamic support—to resuscitate the patient. Blood culture should be done and broad-spectrum antibiotics should be started. Prompt surgical intervention is done by laparotomy. The other method is endovascular repair of the aorta (EVAR) which involves placement of an aortic stent graft under fluoroscopic guidance via bilateral femoral artery cutdowns
	Upper endoscopy—in a hemodynamically stable patient in appropriate setting	

DCF	CT scan, CTA, MRI of the abdomen, or cavography	Resuscitation of the patient by aggressive hemodynamic support followed by surgery. Other option is placement of an endovascular stent into the inferior vena cava as a temporary measure to stop the bleeding with an intention of doing definitive surgery on a later date when the patient is more stable. Intravenous broad-spectrum antibiotics are started preoperatively and continued postoperatively for about a month followed by oral antibiotics for about 2 months. Antibiotics are modified depending on the culture report

**Table 3:** Key studies of GI fistulas.

Study	Key findings	Reference no.
P. E. Fischer, T. C. Fabian, L. J.Magnotti, et al. 2009. “A Ten-Year Review of Enterocutaneous Fistulas After Laparotomy for Trauma,” *The Journal of Trauma, Injury, Infection, and Critical Care* 67, no. 5 (November): 924-928, https://doi.org/10.1097/TA.0b013e3181ad5463	1.9% of trauma laparotomy patients had ECF. Spontaneous closure: low output fistula—55%. High output fistula—31%	[2]
I. González-Pinto and E. M. González. 2001.“Optimising the Treatment of Upper Gastrointestinal Fistulas,” *Gut* 49, no. 4 (December): 422-431, https://doi.org/10.1136/gut.49.suppl_4.iv21	Three-stage strategy for management: diagnosis, conservative treatment (parenteral nutrition, bowel rest, infection control), and surgical intervention if needed. Somatostatin-14 and octreotide reduce fistula output and closure time	[3]
F. F. Willingham and J. M. Buscaglia. 2015. “Endoscopic Management of Gastrointestinal Leaks and Fistulas,” *Clinical Gastroenterology and Hepatology* 13, no. 10 (October): 1714-1721, https://doi.org/10.1016/j.cgh.2015.02.010	Endoscopic management includes stent placement, clip closure, endoscopic suturing, and tissue sealants. Multidisciplinary approach is essential	[102]
C. Levy and W. J. Tremaine. 2002. “Management of Internal Fistulas in Crohn's Disease,” *Inflammatory Bowel Diseases* 8, no. 2 (March): 106-111, https://doi.org/10.1097/00054725-200203000-00007	Internal fistulae in Crohn's disease require multimodal assessment (endoscopy, MRI). Anti-TNF therapies are effective, but many cases need surgical intervention	[136]
M. Falconi and P. Pederzoli. 2001. “The Relevance of Gastrointestinal Fistulas in Clinical Practice: A Review,” *Gut* 49, no. 4 (December): 42-110, https://doi.org/10.1136/gut.49.suppl_4.iv2	Postoperative fistulas are common, with significant complications. Improved care has reduced mortality but not incidence	[1]
	Somatostatin and octreotide increase the likelihood of closure of enterocutaneous fistula. Both are beneficial in reducing the time to fistula closure. Neither has any effect on reducing mortality	[85]
L. R. Rábago, N. Ventosa, J. L. Castro, J. Marco, N. Herrera, F. Gea. 2002. “Endoscopic Treatment of Postoperative Fistulas Resistant to Conservative Management Using Biological Fibrin Glue,” *Endoscopy* 34, no. 8 (August): 632-638, https://doi.org/10.1055/s-2002-33237	Endoscopic fibrin glue treatment achieves a very high success rate, without complications and at a lower cost. It can probably reduce the hospital stay, and avoid some unnecessary surgical interventions	[100]

## Data Availability

The data used to support the findings of this study are available from the corresponding author upon request.
